# Comparison of Multi-Drug Resistant Environmental Methicillin-Resistant *Staphylococcus aureus* Isolated from Recreational Beaches and High Touch Surfaces in Built Environments

**DOI:** 10.3389/fmicb.2013.00074

**Published:** 2013-04-04

**Authors:** Marilyn C. Roberts, Olusegun O. Soge, David No

**Affiliations:** ^1^Department of Environmental and Occupational Health Sciences, University of WashingtonSeattle, WA, USA; ^2^Department of Global Health, University of WashingtonSeattle, WA, USA

**Keywords:** MRSA, environmental, recreational beaches, high touch surfaces, USA300

## Abstract

Over the last decade community-acquired methicillin-resistant *Staphylococcus aureus* (MRSA) has emerged as a major cause of disease in the general population with no health care exposure or known classical risk factors for MRSA infections. The potential community reservoirs have not been well defined though certain strains such as ST398 and USA300 have been well studied in some settings. MRSA has been isolated from recreational beaches, high-touch surfaces in homes, universities, and other community environmental surfaces. However, in most cases the strains were not characterized to determine if they are related to community-acquired or hospital-acquired clinical strains. We compared 55 environmental MRSA from 805 samples including sand, fresh, and marine water samples from local marine and fresh water recreational beaches (*n* = 296), high touch surfaces on the University of Washington campus (*n* = 294), surfaces in UW undergraduate housing (*n* = 85), and the local community (*n* = 130). Eleven USA300, representing 20% of the isolates, were found on the UW campus surfaces, student housing surfaces, and on the community surfaces but not in the recreational beach samples from the Northwest USA. Similarly, the predominant animal ST133 was found in the recreational beach samples but not in the high touch surface samples. All USA300 isolates were multi-drug resistant carrying two to six different antibiotic resistance genes coding for kanamycin, macrolides and/or macrolides-lincosamides-streptogramin B, and tetracycline, with the majority (72%) carrying four to six different antibiotic resistance genes. A surprising 98% of the 55 MRSA isolates were resistant to other classes of antibiotics and most likely represent reservoirs for these genes in the environment.

## Introduction

*Staphylococcus aureus* is part of the normal flora and can be found in the anterior nares as well as on the skin, axilla, perineum, and pharynx. It has been estimated that 25–35% of healthy humans in the general community have *S. aureus* in their anterior nares (Grundmann et al., 2006). However the range varies depending on the population examined and when the study was done. It is now thought that ∼20% of population almost always carries *S. aureus*, another 20% rarely carries *S. aureus*, and the remaining 60% of the population is intermittently colonized (Kluytmans et al., 1997).

Methicillin-resistant *S. aureus* (MRSA) was first identified over 50 years ago and has become a major nosocomial and community pathogen. MRSA strains are *S. aureus* that have a *mecA* gene which codes for a unique penicillin-binding protein that has decreased affinity for β-lactams. This protein allows for cell growth in the presence of penicillins and other β-lactam antibiotics, which are the antibiotics of choice for staphylococcal skin and soft tissue infections (Eady and Cove, 2003; Grundmann et al., 2006). Over the last decade community-acquired MRSA (CA-MRSA) causing primarily skin and soft tissue infections has emerged as a major cause of disease in the general population with no health care exposure or known classical risk factors for MRSA infections around the world (King et al., 2006). In 2005 there were ∼19,000 deaths due to MRSA in hospitalized USA patients. More importantly three fourth of the US MRSA infections were in people that had not been previously hospitalized, had no contact with hospitals, and were part of the general community (Klevens et al., 2007). CA-MRSA morbidity and mortality per 100,000 people have been estimated at 4.6 and 0.5, respectively (Klevens et al., 2007). CA-MRSA strains initially from community patients are now entering the hospital environments to become the predominant nosocomial MRSA isolates (Popovich et al., 2008). In North America, most CA-MRSA disease is due to a single strain, USA300, which produces a number of toxins and has the ability to cause skin and soft tissue infections in otherwise healthy individuals and appears to be more robust on fomites (Desai et al., 2011). In other geographical locations, CA-MRSA can be a variety of different strains (Nimmo and Coombs, 2008; Bartels et al., 2009). Originally CA-MRSA causing disease was less likely to be resistant to other antimicrobial agents than MRSA strains isolated from the high-antibiotic selection pressure of the hospital environment (Eady and Cove, 2003).

*Staphylococcus aureus* and MRSA can be transmitted from people-to-people, from fomites-to-people, and from air-to-people (Huang et al., 2006; Zuckerman et al., 2009). People colonized or infected with *S. aureus* or MRSA shed into their environments contaminating surfaces and fomites at concentrations sufficient for survival for extended periods of time in the environment which should allow for transfer to skin, clothing, and other fomites (Boyce et al., 1997; Otter et al., 2011).

Environmental reservoirs associated with healthcare settings and closed communities such as daycares, schools, prisons, and sport teams have been examined (Centers for Disease Control and Prevention, 2001; Eady and Cove, 2003; Nguyen et al., 2005; Roberts et al., 2011a,b,c). *S. aureus* and MRSA contamination in the marine environment have been examined including shedding from recreational bathers, untreated wastewater, and urban runoff (Selvakumar and Borst, 2006; Elmir et al., 2007; Börjesson et al., 2009; Plano et al., 2011). Over the past several years, *S. aureus* and MRSA have been isolated from marine water, stream water, and intertidal sand samples from beaches in California (Goodwin and Pobuda, 2009), Florida (Abdelzaher et al., 2010), Hawaii (Tice et al., 2010; Viau et al., 2011), and the Pacific Northwest (Soge et al., 2009). MRSA spatial distribution or strain characterization was not addressed in the studies by Goodwin and Pobuda (2009) in California and Tice et al. (2010) in Hawaii. A more recent study sampled fresh water streams draining into coastal beaches on O’ahu Hawaii found *S. aureus* (Viau et al., 2011). Temporal and spatial distribution of pathogenic microorganisms including *S. aureus* was examined in a Florida study (Abdelzaher et al., 2010), however none of the presumptive *S. aureus* isolates were biochemically confirmed as *S. aureus*. The study by Soge et al. (2009) identified MRSA and/or *S. aureus* isolated in 2008, from 6 of 10 marine beaches from the Pacific Northwest, however the number of samples taken was limited. That study found that the MRSA isolates were related to strains previously associated with hospital-acquired MRSA (five SSC*mec* type I, and one non-typeable and ST types 30, 45, 59, 1405), and the *S. aureus* isolates (ST15, ST30, ST59) suggested that they are related to hospital-acquired MRSA. ST5, ST30, ST45, ST59 were all major clonal complexes found in the United States (Schwalm et al., 2011).

In this current paper we compare 55 environmental MRSA isolates, including 11 USA300, collected from 805 samples taken from fresh and marine beaches, high touch surfaces within the community and the University of Washington. This represents the first comparison of characterized MRSA isolates from different natural and built environments.

## Materials and Methods

### Bacterial isolates

All samples from recreational marine or fresh water beaches, and swabs from the environmental surfaces were enriched in Bacto^®^m *Staphylococcus* broth (1.5×) (Difco Laboratories, Sparks, MD, USA) supplemented with a final concentration of 75 μg/L polymyxin B and 0.1% potassium tellurite (Sigma Co., St. Louis, MO, USA) and 50 ml of media was used for wash clothes used for sampling the washing machines as previously described (Roberts et al., 2011c; Levin-Edens et al., 2012). All samples were incubated in 5% CO_2_ at 36.5°C until turbid and black. Positive samples were diluted and plated onto Bacto^®^ *Staphylococcus* Medium 110 (Difco) supplemented with 10 μg/ml methicillin and 0.01% potassium tellurite and Bacto^®^ Mannitol Salts Agar (Difco), and the resulting strains were biochemically verified as *S. aureus* and confirmed as *mecA* positive by PCR assay as previously described (Roberts et al., 2011c; Levin-Edens et al., 2012). Twenty-two MRSA isolated from fresh water draining into recreational marine or fresh water beaches, seven MRSA isolates collected from sand, and two MRSA isolates collected from marine water identified from sand, fresh, and marine water samples from local marine and fresh water recreational beaches (*n* = 296) in 2010 (Levin-Edens et al., 2012) were included in the study. An additional 24 MRSA isolates from frequently touched 509 non-hospital environmental surfaces taken at a large university (*n* = 294), student homes (*n* = 85), and local community sites (*n* = 130) collected 2009–2010 (Roberts et al., 2011c) were included in the comparison (Table 1).

**Table 1:** **Genotypic characteristics of MRSA isolates from recreational beaches and built environmental high touch surfaces**.

Isolate	Source	SCC*mec* type^a^	MLST^b^	PFGE type	USA300	Antibiotic resistance genes^c^
***n* = 31**	**Recreational beaches 2010**					
401	Fresh water	IV	5	L		*tet*(M), *tet*(K), *erm*(C), *msr*(A), *aadD*
PC3	Fresh water	NT	6			*tet*(M), *tet*(K), *msr*(A), *aadD*
1012	Fresh water	NT	6			*tet*(M), *tet*(K), *msr*(A), *aadD*
111	Sand	IV	8	K		*erm*(C), *msr*(A), *aadD*
3mid	Sand	IV	15	G		*tet*(M), *tet*(K), *msr*(A), *aadD*
118	Fresh water	IV	15			*tet*(M), *tet*(K), *erm*(C), *msr*(A), *aadD*
603	Sand	IV	30	H		*tet*(M), *tet*(K), *erm*(C), *msr*(A), *aadD*
1112 (2)	Fresh water	II	45	M		*erm*(A), *aadD*
Seep1	Marine sand	IV	88			*tet*(M), *tet*(K), *msr*(A), *aadD*
257	Marine sand	IV	97	N		*tet*(K), *erm*(C), *msr*(A), *aadD*
302	Marine water	IV	109	F		*tet*(M), *tet*(K), *erm*(A), *msr*(A), *aadD*
308	Marine water	IV	109	F		*tet*(M), *erm*(A), *erm*(C), *msr*(A), *aadD*
361	Sand	IV	109	F		*tet*(M), *erm*(A), *erm*(C), *msr*(A), *aadD*
1112	Fresh water	I	133	B		None
244	Fresh water	IV	133	A		*erm*(C), *msr*(A), *aadD*
248	Fresh water	IV	133	A		*tet*(K), *msr*(A), *aadD*
252	Fresh water	IV	133	B		*tet*(K), *erm*(C), *msr*(A), *aadD*
125	Fresh water	IV	133	C		*tet*(K), *erm*(C), *msr*(A), *aadD*
909	Fresh water	IV	133	D		*tet*(K), *erm*(C), *msr*(A), *aadD*
910	Sand	IV	133	D		*tet*(K), *erm*(C), *msr*(A), *aadD*
512	Fresh water	IV	133	E		*tet*(K), *erm*(C), *msr*(A), *aadD*
824	Fresh water	NT	133			*tet*(K), *erm*(C), *msr*(A), *aadD*
515	Fresh water	IV	1875			*tet*(K), *erm*(C), *msr*(A), *aadD*
526	Fresh water	IV	1875			*tet*(M), *tet*(K), *msr*(A), *aadD*
813	Fresh water	IV	1875			*tet*(M), *tet*(K), *erm*(C), *msr*(A), *aadD*
823	Fresh water	NT	1875			*tet*(M), *tet*(K), *erm*(C), *msr*(A), *aadD*
1113	Fresh water	I	1956	J		None
1124	Fresh water	I	1956			*aadD*
827	Fresh water	IV	1956	I		*tet*(K), *erm*(C), *msr*(A), *aadD*
1017	Fresh water	NT	2049			*erm*(C), *aadD*
1019	Fresh water	NT	2049			*tet*(K), *erm*(C), *msr*(A), *aadD*
***n* = 10**	**Student homes 2010**					
S3–23	TV remote	IV	5	3	Yes	*tet*(M), *tet*(K), *msr*(A), *aadD*
S9–18	Bathroom floor	IV	5	3	Yes	*tet*(M), *msr*(A), *aadD*
S5–28	Bathroom light switch	IV	5	3	Yes	*tet*(M), *tet*(K), *aadD*
T4–38	Washing machine	IV	5	5		*tet*(M), *tet*(K), *msr*(A), *aadD*
J-5	Dorm water fountain	NT	8	1		None
R501	Couch	IV	8	4	Yes	*tet*(M), *msr*(A), *aadD*
R502	Toilet flush handle	IV	8 (931)^d^	4	Yes	*tet*(M), *tet*(K), *msr*(A), *aadD*
R509	Bathroom door knob	IV	8 (931)^d^	4	Yes	*tet*(M), *tet*(K), *msr*(A), *aadD*
A3–6	Microwave touchpad	IV	45	2		*tet*(M), *msr*(A), *aadD*
A4–3	Couch	IV	45	2		*tet*(M), *msr*(A), *aadD*
***n* = 10**	**University campus 2010**					
3–36	Bathroom	IV	5	3	Yes	*erm*(C), *msr*(A)
20	ATM	IV	5	3	Yes	*tet*(M), *tet*(K), *erm*(A), *erm*(C), *msr*(A), *aadD*
3–22	Bathroom	NT	5	12		*tet*(M)
3–2	Elevator button	I	8	9		*tet*(M), *erm*(C), *msr*(A)
8–509	Locker handle	IV	8	8	Yes	*tet*(M), *tet*(K), *msr*(A), *aadD*
26	ATM	IV	30	6	Yes	*tet*(M), *tet*(K), *erm*(A), *erm*(C), *msr*(A), *aadD*
36	ATM	I	45	7		*tet*(M), *tet*(K), *erm*(A), *erm*(C), *aadD*
3–6	Bathroom floor	NT	97	11		*tet*(M)
3–8	Elevator button	NT	97	10		*tet*(M)
3–10	Study lounge floor	NT	97	10		*tet*(M), *erm*(C)
***n* = 4**	**Community 2009–2010**					
40	ATM	NT	5	3	Yes	*tet*(M), *msr*(A)
55	ATM	IV	8	13		*tet*(M), *erm*(C), *msr*(A), *aadD*
5–3	Library computer touch screen	NT	30	14		*tet*(K), *aadD*
5–4	Library computer touch screen	IV	30	14		*tet*(M), *tet*(K), *msr*(A), *aadD*

*^a^SCC*mec* I–V was tested if not one of these types, listed as non-typeable (NT); ^b^MLST typing using seven housekeeping genes; ^c^Kanamycin resistance gene, *aadD*; macrolide, lincosamide-streptogramin B resistance genes *erm*(A), *erm*(B), *erm*(C), and *msr*(A) gene and tetracycline resistance genes *tet*(M) and *tet*(K) were tested; ^d^ST931 has recently been reassigned as ST8 due to revision of the *gmk* locus (Larsen et al., 2012)*.

### Characterization of MRSA strains

The SCC*mec* typing was done by multiplex PCR assay for types I–V using positive controls. SCC*mec* typing classifies the SCC*mec* elements based on their structural differences and has been used to discriminate MRSA strains and define MRSA clones^1^. Those that were not SCC*mec* type I–V were labeled as non-typeable (NT) (Soge et al., 2009). The MLST was previously determined by PCR and sequencing of the PCR products for the seven housekeeping genes (Soge et al., 2009). Alleles were assigned by a comparison of their sequences with the corresponding loci in the *S. aureus* MLST database and combined into an allelic profile with unique sequence type (ST)^2^. Drug resistance genes including kanamycin resistance gene, *aadD*, macrolide-lincosamide-streptogramin B resistance genes *erm*(A), *erm*(B), *erm*(C), and macrolide-streptogramin B resistant *msr*(A) gene, and tetracycline resistance genes *tet*(M) and *tet*(K) were previously determined by PCR assays (Soge et al., 2009). These specific antibiotic resistance genes were selected because they have previously been identified in MRSA isolates or have been associated with SCC*mec* elements (Soge et al., 2009; McDougal et al., 2010; Roberts et al., 2011a,b,c; Levin-Edens et al., 2012). Pulsed-field gel electrophoresis (PFGE) analysis was previously done and isolates were compared to four clinical MRSA USA300 strains used as positive controls to determine whether the environmental isolates were related to USA300 clone (McDougal et al., 2010).

## Results and Discussion

### Distribution of MRSA isolates

Methicillin-resistant *Staphylococcus aureus* was isolated from 10/85 (11.8%) university student homes surfaces and five (63%) of the eight undergraduate student homes had MRSA positive samples. The percentage of MRSA positive samples, which include only samples that yielded MRSA isolates and was a portion of the samples which were contaminated with *S. aureus*, in our university study was 10-fold higher than the 1.1% MRSA positive found from 1,320 surface samples taken from 35 homes in the Boston area (Scott et al., 2009). Our study had higher number of MRSA positive homes compared to the 9/35 (25.7%) of the homes that were contaminated with MRSA in the Boston study. In the university study, eight (9.4%) methicillin-susceptible *S. aureus* (MSSA) isolates from 3 (37.5%) homes versus the Boston study which found MSSA contaminated surfaces in 34 (97.1%) of 35 Boston homes. However, the demographics of the household members did vary between the current and previous study as did the number of samples per home and the number of homes tested (Scott et al., 2009). Thirty-one (10.5%) of the 296 recreational beach samples were positive for MRSA with 22 isolates (71%) coming from fresh water streams running into the marine and freshwater beaches. Fourteen (4.7%) of the recreational beach samples were *S. aureus* positive with 12 (85.7%) of the isolates coming from the fresh water stream samples draining into the marine beaches. A recent study in Hawaii found *S. aureus* in 19 (86.4%) of 22 fresh water streams with 42 (48.8%) of the 86 samples positive for *S. aureus* and one (5.2%) sample positive for MRSA (Viau et al., 2011). The source of the isolates was not identified nor was potential environmental sources examined or isolates further characterized in the Viau et al. (2011) study. Ten (3.4%) out of 294 university surface samples were MRSA positive, while nine (3.1%) were MSSA. This compares to an earlier study which found no MRSA from 70 high touch surfaces in a large urban US university, though samples from keyboards, telephone mouthpieces, and an elevator button were positive for *S. aureus* (Brooke et al., 2009). Four (3.1%) out of 130 community samples were MRSA positive, and one (0.8%) sample was MSSA positive. The closest community studies for comparison have been from public transport system. A London study found 9 (8%) of the 112 samples taken from hand-touch surfaces in the public transport system and in public areas of a hospital were positive for *S. aureus* but no MRSA was isolated (Otter and French, 2009). A recent study from Japan where environmental samples were collected from 2008 to 2010 found 8 (2.3%) of the 349 trains examined were positive for MRSA (Iwao et al., 2012).

Eleven (20%) of the 55 isolates had PFGE patterns that indicated that they were USA300 strains, the most prevalent community-acquired MRSA in the United States (Tenover and Goering, 2009). However in other parts of the world other strains of CA-MRSA have been identified (Nimmo and Coombs, 2008; Bartels et al., 2009). The USA300 isolates were not evenly distributed in all the environments sampled since all 11 came from samples in built environmental high touch surfaces (student housing 6/10; university 4/10; and community 1/4). Three different MLST types were associated with the USA300 PFGE pattern including ST5, ST8, and ST30 (Table 1).

### Drug resistance characterization

Originally, CA-MRSA isolates were less likely to be resistant to other classes of antibiotics, however over the last decade USA300 isolates have become increasingly resistant to fluoroquinolones with 54% resistant by 2006 (Eady and Cove, 2003; McDougal et al., 2010). An early collection of 187 MRSA strains identified 30 USA300 isolates that were multiple-drug resistant and carried macrolide [*msr*(A) gene], and/or macrolide, lincosamide-streptogramin B [*erm*(A), *erm*(C) genes], and/or tetracycline resistance gene [*tet*(K)], and/or levofloxacin resistance due to gyrase mutations (Tenover et al., 2006). While a more recent study of 823 USA300 clinical isolates from the Centers for Disease Control and Prevention (CDC) collection found that 43% of the isolates were resistant to erythromycin and carried the *msr*(A) gene and 43% were resistant to erythromycin due to the presence of the *msr*(A) gene plus levofloxacin resistance due to gyrase mutations, and 8.4% were resistant to tetracycline due to presence of the *tet*(K) gene of which some also carried the *msr*(A) gene and/or the gyrase mutations, leaving just 5.6% susceptible to other classes of antibiotics examined (McDougal et al., 2010). However few groups have characterized the antibiotic resistance patterns of MRSA isolates found in the environment besides the study of Soge et al. (2009). In that study, five MRSA SCC*mec* type I isolates were resistant to one to four classes of antibiotics and all five carried the *erm*(A) gene, one carried both *tet*(K) and *tet*(M) genes, and one carried the *tet*(K) and the other carried the *tet*(M) tetracycline resistance genes. One of the MRSA isolates was able to transfer the *tet*(M) and *erm*(A) genes in mating experiment suggesting that MRSA strains could be environmental reservoirs for antibiotic resistance genes. In addition, 17/21 (81%) of the methicillin-resistant coagulase negative *Staphylococcus* spp. (MRCoNS) were resistant to one to five other classes of antibiotics and carried combinations of *erm*(B), *erm*(C), *msr*(A), *tet*(K), and/or *tet*(M) genes suggesting that MRCoNS strains could also be environmental reservoirs for mobile antibiotic resistance genes. In contrast, none of the environmental MSSA isolates carried any of the resistance genes examined.

Fifty-two (95%) isolates in the current study carried a variety of other antibiotic resistance genes and were multi-drug resistant, including all 11 USA300 isolates. Only three (5.5%) of the 55 MRSA isolates were negative by PCR for all seven antibiotic resistance genes examined and represented three different MLST types (Table 1). The most commonly found gene *aadD* was identified in 45 (82%) isolates, 42 (76%) isolates were positive for *msr*(A), 46 (84%) isolates positive for either *tet*(K) and/or *tet*(M), 27 (49%) isolates positive for *erm*(C), and 7 (13%) isolates positive for *erm*(A). None of the MRSA isolates carried the *erm*(B) gene. Iwao et al. (2012) examined susceptibilities of eight MRSA isolates from the public trains to nine other non-β-lactam antimicrobial agents and all were resistant to two to seven of the agents tested though specific resistance genes present were not determined. Seven (87.5%) of the eight strains resistant to kanamycin likely carried the *aadD* gene, which is similar to the 82% found in the current study, while 4 (50%) were macrolide resistant and 3 (37.5%) were clindamycin resistant. Three (75%) of the four Japanese isolates identified as ST8 CA-MRSA were resistant to gentamicin and kanamycin.

Nineteen (35%) of the MRSA isolates were positive for one to three different antibiotic resistance genes, and included 8 (57%) of the 13 MLST types from all the different environments and five of the USA300 strains. While 33 (60%) of the MRSA isolates carried four to six of the genes analyzed and included isolates from all the different environments with all the 13 MLST types and 6 of the 11 USA300 included in this group (Table 1).

Twenty-two (40%) isolates carried four different antibiotic resistance genes and were isolated from the recreational beaches (*n* = 15), student homes (*n* = 4), university sites (*n* = 1), and community sites (*n* = 2). Ten of the isolates carried *aadD*, *erm*(C), *msr*(A), and *tet*(K) genes and included ST97 (*n* = 1), ST133 (*n* = 6), ST1875 (*n* = 1), ST1956 (*n* = 1), and ST2049 (*n* = 1), and eight were SCC*mec* IV and two NT. Eleven isolates carried *aadD*, *msr*(A), *tet*(K), and *tet*(M) genes and included ST5 (*n* = 2), ST6 (*n* = 2), ST8 (*n* = 3), ST15 (*n* = 1), ST30 (*n* = 1), ST88 (*n* = 1), and ST1875 (*n* = 1). Four (36%) of the 11 MRSA were USA300 strains by PFGE analysis.

The highest number of resistance genes was found in two USA300 isolates, from the University of Washington sites. They were positive for 6 genes [*aadD*, *erm*(A), *erm*(C), *msr*(A), *tet*(K), and *tet*(M)], included ST5 and ST30, and both isolates carried the SCC*mec* IV element. Nine (16%) isolates which carried five different antibiotic resistance genes were isolated from the recreational beaches *n* = 8, and university sites *n* = 1. Five isolates were positive for *aadD*, *erm*(C), *msr*(A), *tet*(K), and *tet*(M) genes, included ST5 (*n* = 1), ST15 (*n* = 1), ST30 (*n* = 1), ST1875 (*n* = 2), and four of the five isolates carried the SCC*mec* IV element. Two SCC*mec* IV, ST109 MRSA isolates from the recreational beaches were positive for *aadD*, *erm*(A), *erm*(C), *msr*(A), and *tet*(M) genes. One other SCC*mec* IV, ST109 isolate from the beach was positive for *aadD*, *erm*(A), *msr*(A), *tet*(K), and *tet*(M) genes (Table 1). One SCC*mec* I, ST45 isolate from the university site was positive for *aadD*, *erm*(A), *erm*(C), *tet*(K), and *tet*(M) genes (Table 1).

### Comparison of SCC*mec* typing and MLST of isolates across different WA environments

Of the 55 MRSA isolates, 35 (63.6%) carried the SCC*mec* IV, while 14 (25.5%) were non-typeable which meant they did not carry SCC*mec* I–V, five (9.1%) carried the SCC*mec* I and one (1.8%) carried the SCC*mec* II (Table 1). In the much smaller Japanese study (Iwao et al., 2012), the SCC*mec* IV was also the most common [4/8 (50%)] with three (37.5%) isolates carrying the SCC*mec* II and one (12.5%) the SCC*mec* I elements. Few other studies of environmental MRSA have SCC*mec* typed their isolates.

There were 13 different MLST types identified from the WA isolates. Nine (69%) of these MLST types have commonly been isolated from humans (see text footnote 2). All 13 of the MLST types were found in MRSA isolated from the WA recreational beaches, five MLST types were found in MRSA isolated from the university, three MLST types (ST5, ST8, and ST45) were found in MRSA isolated from student home surfaces, and three MLST types (ST5, ST8, and ST30) were found in MRSA from the community surfaces. Two new MLST types ST1875 (*n* = 4), three SCC*mec* IV and one NT, and ST2049 (*n* = 2) both NT, were identified in recreational beach samples. The ST2049 is closely related to ST1962 which has previously been identified in squirrels as has ST1956 (see text footnote 2), both of which were found in the recreational beach samples. Interestingly, two of the three ST1956 isolates carried SCC*mec* I, more commonly associated with hospital-acquired MRSA. ST1875 (SCC*mec* IV, *n* = 3; NT, *n* = 1) is closely related to recently identified human ST1176 (CC5) from Brazil (Carmo et al., 2011) and a hospital-acquired ST1176 MRSA strain 2929B reported from Boston, MA, USA and thus could be of human origin (see text footnote 2).

Twenty-six (47%) of the MRSA belonged to three of the 13 MLST types and included nine isolates of ST133 (CC133), nine isolates of ST5 (CC5), and eight isolates of ST8 (CC8). ST5 and ST8 were found in all four environments (Table 1). All nine ST133 isolates were from both the marine and fresh recreational beaches and surrounding fresh water streams draining into the recreational beaches at multiple time points (Levin-Edens et al., 2012). Based on difference in PFGE patterns the nine isolates represented at least five different strains (Table 1). Seven of the MRSA strains representing all five different PFGE patterns carried the SCC*mec* IV element, one carried SCC*mec* I element, and one carried an element that was different than the I–V. Smyth et al. (2009) stated that *S. aureus* ST133 is an ungulate-animal-specific genotype with no association with humans reported in the literature. The ST133 was the most common lineage reported in *S. aureus* isolates collected from Danish small ruminants (65% of 179 sheep and 55% of 17 goats) (Eriksson et al., 2013). ST133 has been isolated from German wild boars (Meemken et al., 2013), and Japanese cats and dogs (Sasaki et al., 2012). More recently a single MRSA ST133 has been identified from 66 MRSA isolates with seven major clonal complexes from nasal swabs collected from patients upon admission to intensive care units at Pennsylvania State University (Schwalm et al., 2011). Thus it is likely that MRSA ST133 is only rarely associated with humans, which correlates with its absence from human built environment surfaces. It is possible that ST133 is associated with common wild and/or pet animals found in urban areas such as Seattle in addition to ungulates and thus explains the wide distribution among the recreational beach areas at multiple time points within the city but its absence from surfaces in built environments in the same geographical area.

The nine ST5 isolates were found at the beaches (*n* = 1), and on the high touch surfaces in the student homes (*n* = 4), university campus (*n* = 3), and community sites (*n* = 1) (Table 1), represented four different PFGE patterns, seven were SCC*mec* IV and two were NT. ST5 has been associated with humans, chickens, turkey, and pork (see text footnote 2). USA100 a common hospital MRSA is ST5 and is part of the major clonal complex which is widely distributed (Schwalm et al., 2011; Iwao et al., 2012; Verghese et al., 2012; Kawaguchiya et al., 2013). ST5 was found in all four WA environments studied (Table 1).

There were eight ST8 isolates and like ST5 found in all environments sampled (recreational *n* = 1; students homes *n* = 4; university *n* = 2; community *n* = 1). Six of the ST8 were SCC*mec* IV isolated at the beaches, student homes, university campus, and community, while ST8 SCC*mec* I was isolated on a university surface and the ST8 NT isolated from student dorm. ST8 has been associated with USA300 clonal type which is now being found in hospital setting (Kawaguchiya et al., 2013) and four of the isolates had PFGE patterns that indicated that they were USA300 strains. ST8 MRSA has also been isolated from chickens, cows, horses, turkey, beef, and pork samples (see text footnote 2). One of the eight isolates was the only MRSA (strain 8-509) in the study to be Panton-Valentine Leukocidin (PVL) positive while the remaining MRSA were all PVL-negative in the WA samples as compared to four of eight MRSA isolates PVL+ in the Japanese study (Iwao et al., 2012).

Other ST types found in humans ST109 (*n* = 3), humans and animals ST15 (*n* = 2), ST45 (*n* = 4), ST88 (*n* = 1), ST97 (*n* = 4), or humans and meat products ST6 (*n* = 2), ST30 (*n* = 4), and ST40 (*n* = 4). ST97 is rarely found in humans and ∼90% of the submissions to the MLST website lists cow milk, bovine mastitis, or pigs (see text footnote 2). ST30 and ST45 belong to major clonal complexes CC30 and CC45 respectively and are often associated with human disease (Simor et al., 2002; Moore et al., 2010; Isobe et al., 2012). The MRSA ST30 clone, found in three of the WA environments, has been predominantly identified in Japanese hospitalized patients since the 1980s (Isobe et al., 2012) and have also been reported in MRSA infections from other parts of the world (Hetem et al., 2012). MRSA ST45, also found in three of the WA environments, is associated with USA600 which has been reported as an epidemic strain in Central European and Canadian hospitals (Witte et al., 1997; Simor et al., 2002) and been implicated in outbreaks of bloodstream infections in Detroit, MI, USA (Abdel-Haq et al., 2009; Moore et al., 2010).

## Conclusion

Unlike recent studies of clinical USA300, all the environmental USA300 isolates in the current study were multi-drug resistant carrying two to six different antibiotic resistance genes. The majority (72%) carried four to six different antibiotic resistance genes and ∼95% of all the other MRSA isolates were multi-drug resistant. Similar results were found in the Japanese study of MRSA isolates from 2008 to 2010 (Iwao et al., 2012). Why there was such a high level of antibiotic resistance in environmental MRSA isolates is certainly not clear but does correlate with our previous small study (Soge et al., 2009). It is unlikely that the level of resistance found in the environmental MRSA isolates is unique to the Seattle, WA area or to the selected environmental surfaces examined. It will also be of interest to know if the environmental MRSA isolates are acquiring their antibiotic resistance genes from other members of the environmental bacterial community or if some of these genes have originated from bacteria in the hospital setting. The data suggests that the environmental MRSA isolates collected in the current study are potential antibiotic resistance gene reservoirs and should be able to act as donors to the microbes and the environmental antibiotic resistance gene pool and potentially contribute to the human microbiome antibiotic resistance gene pool.

A major difference between MRSA isolates isolated from WA recreational beaches and freshwater streams versus other WA environments is that we found no isolates related to USA300 by PFGE, though MLST type associated with USA300 and SCC*mec* IV were present. In contrast, in the built environments, USA300 MRSA isolates comprised 25–60% of the total number of MRSA isolates recovered (Table 1). Another major difference was that ST133 which has until recently thought to be exclusively an animal clone was one of the most commonly isolated ST type from recreational beaches 9/31 (29%) but was absent from the built environmental sites.

From all environmental sites, SCC*mec* IV was the most common 36/55 (66%) which is comparable with what is occurring in the health care setting with SCC*mec* IV replacing other SCC*mec* types (Healy et al., 2004; D’Agata et al., 2009). The most diversity of MLST types were found in the recreational beaches but this could be due to the larger number of different MRSA isolates included. There are a limited number of studies that have characterized environmental MRSA. The recreational beach study of Seifried et al. (2007) identified 18 USA300 isolates from recreational seawater in Hawaii. These isolates represented eight different *spa* types, at least three different SCC*mec* types and a variety of PFGE types. We did not find USA300 in our recreational samples but did find significant diversity of the MRSA strains as was found in Hawaii. Another study in California suggested that beachgoers could be a possible source of MRSA and *S. aureus* isolated in sand and seawater (Goodwin et al., 2012) though the isolates were not characterized to verify this hypothesis. Thus the differences we have found between MRSA strains isolated at the three recreational beaches in the Seattle and the built environmental surfaces may vary geographically with less differences between recreational beaches isolates taken from California, Hawaii or where the water is warmer and many more people swim. However it is clear that more studies characterizing the MRSA isolated in environmental setting need to be done to allow better understanding of the potential risk involved with environmental contact with MRSA and/or *S. aureus* contaminated community high touch surfaces and recreational beaches.

## Conflict of Interest Statement

The authors declare that the research was conducted in the absence of any commercial or financial relationships that could be construed as a potential conflict of interest.

## Footnotes

^1^http://www.staphylococcus.net/

^2^http://saureus.mlst.net/
